# Long-Term Hemostatic and Endothelial Dysregulation Associated with Cardiovascular Events in Survivors of COVID-19 Previously Admitted to the ICU

**DOI:** 10.3390/ijms26146854

**Published:** 2025-07-17

**Authors:** Raquel Behar-Lagares, Ana Virseda-Berdices, Óscar Martínez-González, Rafael Blancas, Óscar Brochado-Kith, Eva Manteiga, Paula Muñoz-García, María Jose Mallol Poyato, Jorge Molina del Pozo, Marcela Homez-Guzmán, María A. Alonso Fernández, Salvador Resino, María Á. Jiménez-Sousa, Amanda Fernández-Rodríguez

**Affiliations:** 1 Unit of Viral Infection and Immunity, National Center for Microbiology (CNM), Health Institute Carlos III (ISCIII), Ctra. de Pozuelo, 28, 28222 Majadahonda, Madrid, Spain; raquelbehar82@gmail.com (R.B.-L.); anavirseda@externos.isciii.es (A.V.-B.); pmunozgar@gmail.com (P.M.-G.); amandafr@isciii.es (A.F.-R.); 2 Centro de Investigación Biomédica en Red en Enfermedades Infecciosas (CIBERINFEC), Health Institute Carlos III (ISCIII), 28029 Madrid, Spain; oscar.brochado@urjc.es; 3 Critical Care Department, Hospital Universitario del Tajo, Av. Amazonas Central, s/n, 28300 Aranjuez, Madrid, Spain; intensivator@yahoo.es (Ó.M.-G.); rafael.blancas@salud.madrid.org (R.B.); mafernandez@salud.madrid.org (M.A.A.F.); 4 Fundación para la Investigación e Innovación Biomédica del Hospital Universitario Infanta Sofía y Hospital Universitario del Henares (FIB HUIS HHEN), Paseo de Europa, 34, 28703 San Sebastián de los Reyes, Madrid, Spain; 5 Faculty of Health Sciences, Universidad Alfonso X el Sabio, Avda. de la Universidad, 1, 28691 Villanueva de la Cañada, Madrid, Spain; 6 Faculty of Health Sciences, Universidad Rey Juan Carlos, Avenida de Atenas s/n, 28922 Alcorcón, Madrid, Spain; 7 Critical Care Department, Hospital Universitario Infanta Cristina, Av. 9 de Junio, 2, 28981 Parla, Madrid, Spain; emaria.manteiga@salud.madrid.org (E.M.); marcelapatricia.homez@salud.madrid.org (M.H.-G.); 8 Emergency Laboratory, Hospital Universitario del Tajo, Av. Amazonas Central, s/n, 28300 Aranjuez, Madrid, Spain; mjose.mallol@salud.madrid.org (M.J.M.P.); jorge.molina@salud.madrid.org (J.M.d.P.)

**Keywords:** SARS-CoV-2, COVID-19, coagulation, sequels, follow-up

## Abstract

Post-acute sequelae of COVID-19 have been associated with an elevated risk of thromboembolism and adverse cardiovascular events (CVEs). We aim to evaluate whether alterations in poorly studied hemostatic and endothelial proteins are associated with CVEs in patients previously admitted to the ICU and evaluated one year post-discharge. We carried out a cross-sectional study involving 63 COVID-19 patients previously admitted to the ICU one year post-discharge. Plasma levels of factor IX (coagulation factor), protein C, protein S (natural anticoagulant), and von Willebrand factor (VWF, an endothelial marker) were measured using a Luminex 200™ analyzer. Generalized linear models (GLMs) were used to assess the association of these coagulation proteins with CVEs and N-terminal pro-B-type natriuretic peptide (NT-proBNP). We found that lower levels of factor IX (*p* = 0.011), protein C (*p* = 0.028), and protein S (*p* = 0.008) were associated with CVEs one year after ICU discharge. Additionally, at the one-year follow-up, we found lower levels of factor IX (*p* = 0.002) and higher levels of VWF (*p* = 0.006) associated with higher levels of NT-proBNP, underscoring the involvement of both hemostatic imbalance and persistent endothelial dysfunction. Our findings revealed a gender-specific pattern of associations with NT-proBNP levels. These findings highlight the significant role of persistent hemostatic imbalance and endothelial dysfunction in the development of cardiovascular abnormalities among COVID-19 survivors discharged from the ICU.

## 1. Introduction

After the acute phase of COVID-19, around 30–80% of patients continue to have symptoms related to SARS-CoV-2 infection up to several months after recovery, which has been termed long COVID [1]. According to the World Health Organization, long COVID is defined as the continuation or development of symptoms 3 months after the initial SARS-CoV-2 infection and the persistence of these symptoms for at least 2 months.

Studies conducted on patients previously admitted to the ICU up to 12 months post-discharge have demonstrated long-term symptoms such as fatigue, dyspnea, pulmonary fibrosis, pathological findings on computed tomography, functional impairment, and even a worse health-related quality of life [2]. An elevated risk of thromboembolism and adverse cardiovascular events (CVEs) has also been linked to long COVID [3,4]. Furthermore, several reports focused on hospitalized patients after discharge have shown the persistence of cardiac symptoms 6–12 months after discharge, such as fatigue, dyspnea, chest pain, and palpitations [5].

NT-proBNP is widely used as a biomarker of heart failure and cardiac dysfunction [6]. Also, it has significant diagnostic and prognostic implications for asymptomatic and early-stage cardiovascular diseases [7,8]. Remarkably, it has been reported that NT-proBNP could independently predict the risk of in-hospital death in patients with severe COVID-19 [9].

SARS-CoV-2 infection is known to induce a prothrombotic state with a characteristic increase in D-dimer [10]. Patients with severe COVID-19 have been shown to have a longer prothrombin time (PT) and activated partial thromboplastin time (aPTT) with maintained high D-dimer levels [11]. D-dimer, coagulation factor VIII, and von Willebrand factor (VWF) levels have been associated with cardiovascular disease risk [12,13]. However, the relationships between other hemostatic and endothelial-related proteins and this condition remain unclear.

Despite the several abnormalities in the coagulation cascade that have been reported in individuals with long-COVID [14], the precise role of specific hemostatic and endothelial proteins, such as factor IX, protein C, protein S, and VWF, in the onset of long-term cardiovascular sequelae of COVID-19 remains unknown. Since VWF reflects endothelial activation, its assessment alongside natural anticoagulants may offer additional insight into vascular dysfunction not captured by conventional coagulation parameters. Furthermore, men and women exhibit different coagulation profiles [15], with women usually showing a higher coagulability status compared to men. However, gender-specific studies on the hemostatic and endothelial profiles of COVID-19 patients are lacking in the current literature, and to our knowledge, no studies have approached gender differences in patients who were admitted to the ICU.

Thus, our objective was to evaluate whether alterations in understudied hemostatic and endothelial proteins are associated with the development of CVEs in patients previously admitted to the ICU one year after hospital discharge, considering potential gender-related differences.

## 2. Results

### 2.1. Clinical and Epidemiological Data

Clinical and epidemiological data are shown in Table 1 (extended information in Table S1). A total of 63 patients were included in the study. The median age on admission was 59.7 (interquartile range (IQR) = 53.2–68.8) years, with a median follow-up time of 15.4 months (IQR = 13.2–20.9). One year after discharge, 9.5% of patients developed a CVE. A significant difference between patients with and without a CVE was observed solely with the use of enoxaparin during hospitalization.

### 2.2. Association Between Hemostatic and Endothelial Proteins and Cardiovascular Events

**Figure 1:**
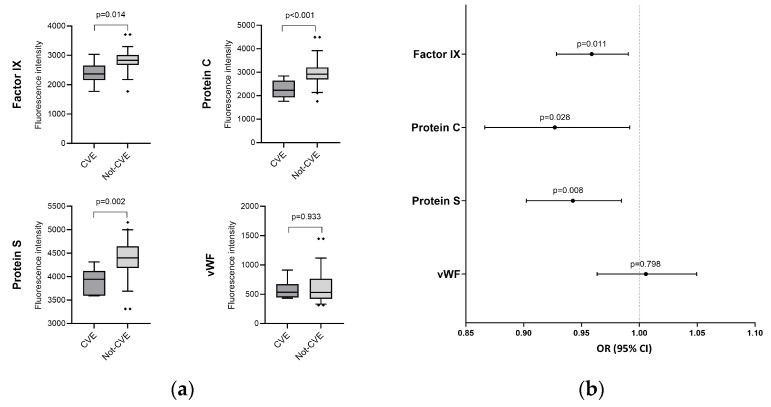
Coagulation protein levels regarding the presence of CVE. (**a**) Boxplots depicting factor IX, protein C, protein S, and VWF levels in patients with (dark gray) and without (gray) cardiovascular events; (**b**) Visual representation of adjusted associations between coagulation protein levels after discharge and the occurrence of cardiovascular events. **Statistics**: Data were calculated using generalized linear models (GLMs) with a binomial distribution. Odds ratio, 95% confidence intervals (lower boundary: 2.5%, upper boundary: 97.5%), and *p*-value are shown. GLMs were adjusted for age, gender, body mass index (BMI), follow-up time after discharge, and whether the patient received anticoagulant therapy after discharge. Covariates were selected through a stepwise approach based on the Akaike Information Criterion (AIC). **Abbreviations**: OR, odds ratio; 95%CI, 95% of confidence interval; *p*, level of significance; VWF, von Willebrand factor; CVE: cardiovascular event.

One year after discharge, patients who developed a CVE at any time after discharge showed lower levels of factor IX (*p* = 0.014), protein C (*p* < 0.001), and protein S (*p* = 0.002) (Figure 1). Similarly, after adjusting for relevant covariates using GLMs, factor IX [aOR = 0.96 (0.93–0.99), *p* = 0.011], protein C [aOR = 0.93 (0.87–0.99), *p* = 0.028], and protein S [aOR = 0.94 (0.90–0.98), *p* = 0.008] remained inversely associated with the presence of a CVE (Figure 1, Table S2). Conversely, no differences in aPTT, INR, and VWF were observed between groups. Unfortunately, due to the limited number of cases in the CVE group, subgroup analysis by gender was not performed.

Note that an additional analysis showed no associations between hemostatic and endothelial proteins on ICU admission and a CVE after discharge (Table 2).

### 2.3. Association Between Hemostatic and Endothelial Proteins and NT-ProBNP Levels by Gender

**Figure 2:**
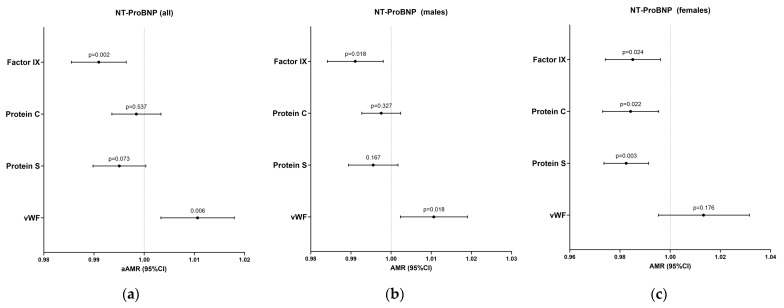
Adjusted associations of coagulation proteins after discharge with NT-proBNP levels for all patients (**a**), males (**b**) and females (**c**). **Statistics**: Data were calculated using generalized linear models (GLMs) with a gamma distribution. AMR, 95% confidence intervals (lower boundary: 2.5%, upper boundary: 97.5%), and *p*-values are shown for both adjusted models. GLMs were adjusted for age, gender, body mass index (BMI), follow-up time after discharge, and whether the patient received anticoagulant therapy after discharge. Covariates were selected through a stepwise approach based on the Akaike Information Criterion (AIC). **Abbreviations**: AMR, arithmetic mean ratio; 95%CI, 95% confidence interval; *p*, level of significance; VWF, von Willebrand factor.

One year after discharge, lower levels of factor IX [aAMR = 0.99 (0.98–0.99), *p* = 0.002] and higher levels of VWF [aAMR = 1.01 (1.01–1.02), *p* = 0.006] were significantly associated with higher levels of NT-proBNP (Figure 2, Table S3). These associations remained for men after stratifying by gender [*p* = 0.018 (VWF) and *p* = 0.018 (factor IX)] (Figure 2, Table S4). Regarding females, factor IX [aAMR = 0.99 (0.97–0.99), *p* = 0.024], protein C [aOR = 0.98 (0.97–0.99), *p* = 0.022], and protein S [aAMR = 0.98 (0.97–0.99), *p* = 0.003] were also significantly associated with higher levels of NT-proBNP (Figure 2, Table S5). Additionally, no significant associations were found between hemostatic and endothelial biomarkers on ICU admission and NT-proBNP levels one year after discharge (Tables S6–S8).

## 3. Discussion

Our findings reveal that patients who developed a cardiovascular event after hospital discharge due to COVID-19 exhibited a distinct hemostatic and endothelial protein profile one year post-discharge. We also identified gender-specific associations with NT-proBNP. To our knowledge, this is the first study to investigate the long-term association between coagulation and endothelial abnormalities and CVE in patients who survived being admitted to the ICU due to COVID-19.

It is well known that SARS-CoV-2 infection induces a procoagulant state, particularly in severe cases [16], during the acute phase. Coagulation abnormalities have been observed even several months after the acute phase, with a prothrombotic state persisting up to 18 months after hospital discharge [10]. These prolonged alterations in hemostatic balance suggest ongoing vascular dysfunction, potentially driven by persistent endothelial injury. Our results may reflect a procoagulant pattern characterized by lower levels of both natural anticoagulants (protein C and protein S) and coagulation factor IX in patients with a CVE. Although several studies have explored factor IX, protein C, and protein S during ICU admission [17,18,19], these proteins have been understudied in the long-term post-discharge period. Fan et al. [4] showed a procoagulant pattern characterized by higher levels of D-dimer and factor VIII levels, and significantly lower antithrombin levels approximately one year after recovery. However, the same study found no differences in protein C and protein S activities in 39 patients compared to the controls. These findings are not necessarily in conflict with ours, as our study focused exclusively on patients admitted to the ICU, while Fan et al. included only seven patients admitted to the ICU in their analysis [4]. Furthermore, our study compared patients admitted to the ICU who developed a CVE with those who did not, in contrast to Fan et al., who compared patients with healthy controls, irrespective of pre-existing cardiovascular pathologies. In addition, Garcia-Larragoiti et al. [20], in a follow-up study, reported higher plasma levels of factor IX in patients with long COVID compared to healthy controls. Unlike our study, which focused specifically on patients admitted to the ICU, this study included COVID-19 patients without specifying their disease severity. However, Garcia-Larragoiti et al. [20] considered not only CVEs, such as tachycardia or arrhythmia, but also other types of post-COVID-19 conditions. These differences may explain the discrepancies with our findings. Nonetheless, larger-scale studies are required to corroborate our findings.

NT-proBNP is a widely recognized biomarker in the assessment of cardiovascular diseases, particularly heart failure [21]. Longitudinal studies showed that the NT-proBNP concentration may remain elevated up to 12 months after infection [22] and that patients with long COVID had significantly higher levels of NT-proBNP compared with asymptomatic patients [23]. In this setting, when investigating the association between hemostatic and endothelial proteins and NT-proBNP, we found that both lower levels of factor IX and increased levels of VWF were linked to higher NT-proBNP concentration one year after discharge. This finding further supports the role of endothelial dysregulation in the cardiovascular complications observed with long COVID. Persistent endothelial activation could be one of the mechanisms underlying the prothrombotic state and elevated cardiovascular risk in this population, although further mechanistic studies are needed to confirm this link.

COVID-19 can trigger a massive inflammatory response with the release of cytokines and chemokines, called a cytokine storm, especially in patients requiring ICU admission [24]. Due to this extensive inflammatory response, endothelial cells release multimers of VWF [25]. Previous studies have confirmed that higher plasma levels of VWF or VWF antigen (VWF:Ag) have been associated with a more severe clinical presentation in the acute phase [26,27] and may remain elevated several months after infection [4]. In addition, some molecules, including VWF, have been shown to be trapped inside microclots of long COVID patients compared to the controls [28]. Likewise, Danielle et al. [26], in a long-term follow-up study, showed that 42.9% (n = 12) of patients with altered VWF levels after 6 months experienced fatigue, 32.1% (n = 9) experienced dyspnea, 17.9% (n = 5) experienced chest pain, and 10.7% (n = 3) reported palpitations, symptoms that could reflect damage to the cardiovascular system. Similarly, Bellone et al. [29] found higher levels of VWF in a small study of 10 gynecological patients with long COVID compared to the controls. These findings are consistent with our results, supporting the association between VWF and CVEs in recovered patients previously admitted to the ICU and reinforcing the concept that VWF plays a pivotal role in the vascular complications observed in the post-COVID-19 period.

Regarding gender, our study showed that lower levels of factor IX, protein C, and protein S were associated with higher levels of NT-proBNP in females, while in males, lower factor IX and higher VWF levels were linked to higher NT-proBNP concentrations. Huang et al. [30] reported that female gender was a risk factor for developing post-acute sequelae of COVID-19 in a longitudinal cohort study (n = 2469). Furthermore, Bai et al. [31] demonstrated that the female gender was associated with the development of post-acute sequelae of COVID-19 at 3 months. The most frequent symptoms described in that study were fatigue and exertional dyspnea, which could be indicative of damage to the cardiovascular system. Consistent with previous evidence, our study suggests that males and females have distinct coagulation profiles, which may impact the development of COVID-19-derived CVEs. These gender-specific findings underlie biological differences in hemostatic and vascular recovery pathways following severe COVID-19.

We observed that the medians of NT-proBNP concentrations (91 pg/mL for the CVE group and 50 pg/mL for the non-CVE group) were within the range of normal values (<125 pg/mL), which would exclude the diagnosis of heart failure [32]. However, Reddy et al. have recently shown that the sensitivity for ruling out heart failure with preserved ejection fraction is improved by an NT-proBNP threshold of 50 pg/mL. They also suggest that accounting for BMI reduces misclassification with the current thresholds [33]. In our analysis, BMI was considered by adjusting the models accordingly. However, the observed associations between higher levels of NT-proBNP and hemostatic/endothelial proteins should be interpreted with caution, highlighting the need for further research.

Regarding additional comparisons between hemostatic and endothelial proteins on ICU admission and CVEs/NT-proBNP levels one-year post-discharge, no associations were found. These findings allowed us to rule out the possibility that baseline alterations in coagulation or endothelial profile were related to higher NT-proBNP levels or CVE development one year later.

Additionally, several considerations should be taken into account for correct data interpretation. Firstly, the limited sample size could have limited the statistical power to detect additional associations. The single-center design and observational nature of the study may also have limited the generalizability of the findings. Secondly, a comparison between baseline hemostatic and endothelial protein levels and baseline NT-proBNP levels on ICU admission would have provided a more comprehensive evaluation of the basal status of patients; however, NT-proBNP data on ICU admission were not available. Thirdly, the coagulation parameters were measured as total protein levels, which do not capture functional activity and therefore limit the ability to assess their biological activity or clinical significance. Additionally, it is important to bear in mind the dual role of NT-proBNP as a marker of both cardiovascular disease and inflammation [22]. Whether these hemostatic and endothelial alterations persist over time or eventually normalize remains an open question, warranting longitudinal studies with extended follow-up.

While these limitations may affect the broader applicability of our results, our study offers meaningful insights into persistent hemostatic and endothelial alterations following severe COVID-19, which may serve as a valuable foundation for future multicenter and longitudinal investigations.

## 4. Materials and Methods

### 4.1. Design and Study Population

A cross-sectional study was conducted on COVID-19 patients admitted to the ICU at Hospital Universitario del Tajo and Hospital Universitario Infanta Cristina between August 2020 and March 2021. The inclusion criteria were as follows: (i) ICU admission related to COVID-19; (ii) assessment of a new CVE (including arrhythmia, stroke, venous thromboembolic disease, or pulmonary thromboembolism) at least one-year post-discharge; (iii) availability of a plasma sample at least one year after discharge; and (iv) availability of clinical data and/or NT-proBNP at least one year after discharge. Patients with pre-existing cardiovascular diseases were excluded. The study protocol was approved by the Ethics Committees of the Institute of Health Carlos III (protocol CEI PI 28_2021-v3) on 24 May 2021, from the Hospital Universitario del Tajo and Hospital Infanta Cristina. Written informed consent was obtained from all patients or their authorized surrogates.

### 4.2. Clinical Data and Samples

An extensive questionnaire was completed both on ICU admission and approximately one year after discharge, in which the clinical and biochemical variables of interest were recorded, including the most relevant symptoms. Data were collected using an electronic case report form (eCRF) built using REDCap electronic data capture tools.

All patients received treatment with enoxaparin during their ICU stay. Patients in the prophylactic-dose group received 40 mg/24 h (<30 mg/24 h if they had renal insufficiency and 60 mg/24 h if their weight was greater than 150 kg). Those in the therapeutic-dose group received 1 mg/kg/12 h of enoxaparin.

Peripheral blood samples were collected in EDTA tubes one year after hospital discharge. The samples were centrifuged the same day of the extraction, and plasma was stored at −80 °C until it was transferred to the National Center for Microbiology for subsequent analysis.

### 4.3. Outcome

The main outcome was the presence of a new CVE one year after hospital discharge, including arrhythmia, stroke, venous thromboembolic disease (VTED), and/or pulmonary embolism. The secondary outcome was the association with NT-proBNP levels.

### 4.4. Measurement of Hemostatic and Endothelial Markers

Plasma concentrations of factor IX, protein C, protein S, and VWF were measured using the Human Coagulation 4-PLEX ProcartaPlex Panel (Invitrogen, Waltham, MA, USA) on a Luminex 200™ analyzer (Luminex Corporation, Austin, TX, USA), following the manufacturer’s protocol. These proteins were selected based on their involvement in coagulation pathways and endothelial-related processes. This multiplex immunoassay quantifies total protein levels rather than their functional activity. The raw fluorescence intensity (FI) values were measured and used (arbitrary units, a.u.) as previously described [34]. The aPTT and international normalized ratio (INR) were measured with the analyzer Siemens CS-2500 System (manufactured by Sysmex Corporation, Kobe, Japan; distributed by Siemens Healthineers, Erlangen, Germany).

### 4.5. Statistical Analysis

For the descriptive study, the Mann–Whitney U test was used to test differences in continuous variables between groups, and the chi-square test and Fisher’s exact test were used for categorical variables when appropriate.

The association between coagulation proteins (independent variables) and CVEs (dependent variable) was analyzed using both unadjusted and adjusted GLMs with binomial distribution. For the adjusted models, covariates were selected through a stepwise approach based on the Akaike Information Criterion (AIC). The covariates used were age, gender, body mass index (BMI), follow-up time post-discharge, and whether the patient received anticoagulant therapy after hospital discharge. Likewise, the association between coagulation proteins (independent variables) and NT-proBNP levels (dependent variable) was assessed using unadjusted and adjusted GLMs with a gamma distribution, employing the same stepwise covariate selection method indicated above.

Furthermore, we performed additional GLMs to examine the relationship between coagulation protein levels on ICU admission and CVE/NT-proBNP levels at one year to exclude the possibility that alterations in coagulation factors on admission are related to CVEs or NT-proBNP levels at the follow-up visit.

A statistically significant association was considered with a *p*-value < 0.05. Statistical software R (v 4.3.0) (www.r-project.org was used for all statistical analyses).

## 5. Conclusions

In conclusion, our data show a distinct hemostatic and endothelial protein profile in survivors of COVID-19 admitted to the ICU who developed a CVE one year after discharge, characterized by reduced levels of natural anticoagulants and factor IX. Gender-specific associations with NT-proBNP were identified: lower levels of these factors correlated with higher NT-proBNP levels in women, while in men, elevated NT-proBNP levels were linked to lower factor IX and increased VWF levels. This novel investigation, focusing on ICU survivors, highlights the persistent impact of severe COVID-19 infection on coagulation and its potential contribution to long-term cardiovascular risk, underscoring the need for continued monitoring in this high-risk population.

## Figures and Tables

**Table 1:** Main clinical and epidemiological characteristics of patients with COVID-19 included in the study.

Characteristics	All Patients
**Demographics**	
No.	63
Age (years)	59.7 (53.2–68.8)
Gender (Male)	43/63 (68.2%)
BMI (kg/m^2^)	31.08 (27.9–34.8)
**Ethnicity**	
Caucasian	50/63 (79.4%)
Hispanic	7/63 (11.1%)
Arabian	3/63 (4.7%)
Other	2/63 (3.2%)
**Comorbidities**	
Non-smoker	45/63 (71.5%)
Ex-Smoker	17/63 (26.9%)
Smoker	1/63 (1.6%)
Arterial hypertension	24/63 (38.1%)
Obesity (BMI > 30)	27/62 (43.5%)
Diabetes	16/63 (25.4%)
**Hepatic Function**	
GOT (UI/L)	36.5 (28.7–57.0)
**Therapy before hospitalization**	
AIIRA	8/63 (12.7%)
ACE	8/63 (12.7%)
Anticoagulant therapy	3/63 (4.8%)
**Oxygen Therapy and Ventilator Support**	
IMV	48/63 (76.2%)
Duration of IMV (days)	9.0 (1.5–23.0)
High-flow nasal cannulas	41/63 (65.1%)
Duration of high-flow nasal cannula therapy (days) (N = 41)	2.0 (0.0–4.0)
**ICU**	
ICU LOS (days)	14.0 (8.0–30)
Prone position	21/63 (33.3%)
**Follow-up**	
Follow-up visit (months)	15.4 (13.2–20.9)

Statistics: Individual characteristics were summarized using standard descriptive statistics: median (interquartile range) for continuous variables and count (percentage) for categorical variables. Differences between groups were tested using the Mann–Whitney U test for continuous variables, and the chi-square test (n ≥ 5) and Fisher’s exact test (n < 5) for categorical variables. Abbreviations: BMI, body mass index; ACE, angiotensin-converting enzyme inhibitors; AIIRA, angiotensin II receptor antagonists; ICU, intensive care unit; ICU LOS, ICU length of stay; IMV, invasive mechanical ventilation; GOT, glutamyl oxaloacetic transaminase.

**Table 2:** Association of coagulation proteins on ICU admission with cardiovascular events one year after discharge.

Coagulation Biomarkers	Unadjusted		Adjusted	
	OR (95%CI)	*p*	aOR (95%CI)	*p*
Factor IX	1.01 (0.82–1.23)	0.962	0.98 (0.72–1.32)	0.881
Protein C	0.95 (0.8–1.12)	0.558	0.94 (0.74–1.21)	0.651
Protein S	0.99 (0.84–1.19)	0.983	0.82 (0.61–1.1)	0.189
VWF	1.17 (0.98–1.41)	0.081	1.06 (0.83–1.34)	0.649
aPTT	1.03 (0.86–1.24)	0.736	1.44 (0.86–2.41)	0.168
INR	1.02 (0.95–1.1)	0.585	1.01 (0.9–1.12)	0.922

Statistics: Data were calculated using generalized linear models (GLMs) with binomial regression. Odds ratio, 95% confidence intervals (lower boundary: 2.5%, upper boundary: 97.5%), and *p*-values are shown for both adjusted and unadjusted models. GLMs were adjusted by age, gender, follow-up time, and body mass index (BMI). Abbreviations: aPTT, activated partial thromboplastin time; INR, international normalized ratio; aOR, adjusted odds ratio; 95%CI, 95% confidence interval; *p*, level of significance; VWF, von Willebrand factor.

## Data Availability

The raw data supporting the conclusions of this article will be made available by the authors on request.

## Supplementary Materials

The following supporting information can be downloaded at: https://www.mdpi.com/article/10.3390/ijms26146854/s1.

## Abbreviations

The following abbreviations are used in this manuscript:

AIC	Akaike Information Criterion
ACE	Adverse cardiovascular events
aAMR	Adjusted arithmetic mean ratio
aPTT	Partial thromboplastin time
BMI	Body mass index
GLM	Generalized linear models
ICU	Intensive care unit
INR	International normalized ratio
IQR	Interquartile range
OR	Odds ratio
PT	Prothrombin time
VTED	Venous thromboembolic disease
VWF	von Willebrand factor
VWF:Ag	VWF antigen
