# Supplementary material for: Long-Term Hemostatic and Endothelial Dysregulation Associated with Cardiovascular Events in Survivors of COVID-19 Previously Admitted to the ICU
Source: Int J Mol Sci. 2025 Jul 17;26(14):6854. doi: 10.3390/ijms26146854 (PMC12294816; doi:10.3390/ijms26146854)
Supplement: Supplementary file 1 [file ijms-26-06854-s001.zip › ijms-3754462-supplementary.pdf]

Supplementary Data Index

Table S1. Clinical and epidemiological characteristics of patients with COVID-19, stratified by cardiovascular events 1

Table S2. Association of coagulation proteins with cardiovascular events one year after ICU discharge. 3

Table S3. Association of coagulation proteins with NT-proBNP levels one year after ICU discharge. 4

Table S4. Association of coagulation proteins with NT-proBNP levels in the male group one year after ICU discharge... 5

Table S5.Association of coagulation proteins with NT-proBNP levels in the female group one year after ICU discharge. 6

Table S6. Association of coagulation proteins at ICU admission with NT-proBNP levels one year after ICU discharge. ... 7

Table S7. Association of coagulation proteins at ICU admission with NT-proBNP levels in the male group one year after ICU discharge. 8

Table S8. Association of coagulation proteins at ICU admission with NT-proBNP levels in the female group one year after ICU discharge. 9

Table S1. Clinical and epidemiological characteristics of patients with COVID-19, stratified by cardiovascular events

Characteristics	All	Not-CVE	CVE	p-value
**Demographics**				
No.	63	57	6	
Age (years)	59.7 (53.2 - 68.8)	61.00 (55.0 - 70.0)	62.0 (59.2 - 67.0)	0.806
Gender (Male)	43/63 (68.2%)	39/58 (68.4%)	4/6 (66.7%)	0.999
BMI (kg/m^2)		29.39 (26.17 - 33.06)	34.13 (32.9 - 34.6)	0.054
**Ethnicity**				
Caucasian	50/63 (79.4%)	45/57 (78.9%)	5/6 (83.3%)	
Hispanic	7/63 (11.1%)	7/57 (12.3%)	0/6 (0.0%)	0.551
Arabian	3/63 (4.7%)	2/57 (3.5%)	1/6 (16.7%)	
Other	2/63 (3.2%)	2/57 (3.5%)	0/6 (0.0%)	
**Comorbidities**				
Non-smoker	45/63 (71.5%)	42/57 (73.7%)	3/6 (50.0%)	
Ex-Smoker	17/63 (26.9%)	14/57 (24.6%)	3/6 (50.0%)	0.400
Smoker	1/63 (1.6%)	1/57 (1.8%)	0/6 (0.0%)	
Arterial hypertension	24/63 (38.1%)	22/57 (38.6%)	2/6 (33.3%)	0.999
Obesity (BMI>30)	27/62 (43.5%)	23/56 (40.4%)	4/6 (66.7%)	0.456
Diabetes	16/63 (25.4%)	15/57 (26.3%)	1/6 (16.7%)	0.981
**Hepatic Function**				
GOT (UI/L)	36.5 (28.7 - 57.0)	40.00 (30.5 - 58.0)	29.00 (25.0 - 51.0)	0.418
**Therapy before hospitalization**				
AIIRA	8/63 (12.7%)	8/57 (14.0%)	0/6 (0.0%)	-
ACE	8/63 (12.7%)	8/57 (14.0%)	0/6 (0.0%)	-
Anticoagulant therapy	3/63 (4.8%)	2/57 (3.5%)	1/6 (16.7%)	0.666
**Treatment during hospitalization**				
Antibiotics	13/63 (20.6%)	13/67 (22.8%)	0/6 (0.0%)	-
Azithromycin	1/63 (1.5%)	1/57 (1.8%)	0/6 (0.0%)	-
Corticoids	13/63 (20.6%)	13/57 (22.8%)	0/6 (0.0%)	-

Characteristics	All	Not-CVE	CVE	p-value
Anticoagulants	56/59 (94.9%)	50/53 (94.3%)	6/6 (100.0%)	0.999
Therapeutic dose of enoxaparin (80-100mg/12h)	6/56 (10.7%)	3/50 (6.0%)	3/6 (50.0%)	**0.009**
**Oxygen Therapy and Ventilator Support**				
IMV	48/63 (76.2%)	43/57 (75.4%)	5/6 (83.3%)	0.999
Duration of IMV (days)	9.0 (1.5 -23.0)	9.00 (0.0 - 20.0)	23.00 (11.0 - 29.7)	0.174
High-flow nasal cannulas	41/63 (65.1%)	36/57 (63.2%)	5/6 (83.3%)	0.592
Duration of high-flow nasal cannula therapy (days) (N=41)	2.0 (0.0 - 4.0)	2.00 (0.0- 3.5)	3.00 (0.0 - 4.0)	0.819
**ICU**				
ICU LOS (days)	14.0 (8.0 -30)	8.0 (0.0 - 16.0)	21.5 (9.0 - 38.5)	0.126
Prone position	21/63 (33.3%)	19/57 (33.3%)	2/6 (33.3%)	0.999
**Follow-up**				
Follow-up visit (months)	15.4 (13.2 - 20.9)	15.48 (13.4 - 21.4)	13.07 (12.5 - 13.9)	0.104
NT-proBNP (pg/mL) (N=48)	50.5 (27.0 -141.5)	50.0 (26.00 - 132.0)	91.00 (50.0 - 164.0)	0.362
Anticoagulant treatment	10/63 (15.9%)	4/57 (7.0%)	6/6 (100.0%)	-
**Cardiovascular events (CVE)**				
Arrhythmia	3/63 (4.8%)	-	3/6 (50.0%)	-
Stroke	2/63 (3.2%)	-	2/6 (33.3%)	-
VTED	1/63 (1.6%)	-	1/6 (16.7%)	-
Pulmonary thromboembolism	2/63 (3.2%)	-	2/6 (33.3%)	-

Statistics: Individual characteristics were summarized using standard descriptive statistics: median (interquartile range) for continuous variables and count (percentage) for categorical variables. Differences between groups were tested using the Mann-Whitney test for continuous variables, and the Chi-square test (n≥5) and Fisher's exact test (n<5) for categorical variables. Abbreviations: BMI, body mass index; ACE, angiotensin converting enzyme inhibitors; AIIRA, angiotensin II receptor antagonists; ICU, intensive care unit; ICU LOS, ICU length of stay; IMV, invasive mechanical ventilation; GOT, glutamyl oxaloacetic transaminase; VTED, venous thromboembolic disease.

Table S2. Association of coagulation proteins with cardiovascular events one year after ICU discharge.

Coagulation biomarkers	***Un-adjusted***		***Adjusted***	
	OR (95%CI)	***p***	***aOR (95%CI)***	***p***
Factor IX	0.96 (0.93-0.99)	**0.012**	0.96 (0.93-0.99)	**0.011**
Protein C	0.96 (0.94-0.99)	**0.003**	0.93 (0.87-0.99)	**0.028**
Protein S	0.97 (0.94-0.99)	**0.011**	0.94 (0.90-0.98)	**0.008**
VWF	0.99 (0.96-1.03)	0.790	1.01 (0.96-1.05)	0.798
aPTT	0.98 (0.94-1.03)	0.516	0.97 (0.91-1.03)	0.302
INR	6.63 (0.85-51.85)	0.071	4.05 (0.47-34.86)	0.202

Statistics: Data were calculated using generalized linear models (GLM) with binomial regression. Odds ratio, 95% confidence intervals (lower boundary: 2.5%, upper boundary: 97.5%) and p-value are shown for both adjusted and unadjusted models. GLMs were adjusted by age, gender, follow-up time and body mass index (BMI). Significant associations are shown in bold. **Abbreviations**: aPTT, activated partial thromboplastin time; INR, international normalized ratio; aOR, adjusted odds ratio; 95%CI, 95% confidence interval; p, level of significance; VWF, von Willebrand factor.

Table S3. Association of coagulation proteins with NT-proBNP levels one year after ICU discharge.

Coagulation biomarkers	***Un-adjusted***		***Adjusted***	
	AMR (95%CI)	***p***	***aAMR (95%CI)***	***p***
Factor IX	0.99 (0.99-1.01)	0.246	0.99 (0.98-0.99)	**0.002**
Protein C	0.99 (0.99-1.01)	0.119	0.99 (0.99-1.01)	0.537
Protein S	0.99 (0.98-0.99)	**0.039**	0.99 (0.99-1.01)	0.073
VWF	1.01 (10.1-1.02)	**0.032**	1.01 (1.01-1.02)	**0.006**
aPTT	1.01 (0.99-1.01)	0.865	0.99 (0.99-1.01)	0.714
INR	1.92 (0.96-3.87)	0.075	1.01 (0.48-2.08)	0.996

Statistics: Data were calculated using generalized linear models (GLM) with gamma distribution. AMR, 95% confidence intervals (lower boundary: 2.5%, upper boundary: 97.5%) and p-value are shown for both adjusted and unadjusted models. GLMs were adjusted by age, gender, age, gender, follow-up time, body mass index (BMI) and anticoagulant therapy at the end of follow-up. Significant associations are shown in bold. **Abbreviations**: AMR, arithmetic mean ratio; aAMR, adjusted AMR; aPTT, activated partial thromboplastin time; INR, international normalized ratio; 95%CI, 95% confidence interval; p, level of significance; VWF, von Willebrand factor.

Table S4. Association of coagulation proteins with NT-proBNP levels in the male group one year after ICU discharge.

Coagulation biomarkers	*Un-adjusted*		*Adjusted*	
	AMR (95%CI)	*p*	*aAMR (95%CI)*	*p*
Factor IX	1 (0.99-1.01)	0.586	0.99 (0.98-1)	**0.018**
Protein C	1 (0.99-1)	0.515	1 (0.99-1)	0.327
Protein S	0.99 (0.98-1)	0.182	1 (0.99-1)	0.167
VWF	1.01 (1-1.03)	0.067	1.01 (1-1.02)	**0.018**
aPTT	1.01 (0.99-1.01)	0.859	1.01 (0.99-1.01)	0.941
INR	1.87 (0.82-4.24)	0.147	1.31 (0.68-2.52)	0.424

Statistics: Data were calculated using generalized linear models (GLM) with gamma distribution. AMR, 95% confidence intervals (lower boundary: 2.5%, upper boundary: 97.5%) and p-value are shown for both adjusted and unadjusted models. GLMs were adjusted by age, gender, age, gender, follow-up time, body mass index (BMI) and anticoagulant therapy at the end of follow-up. Significant associations are shown in bold. **Abbreviations**: AMR, arithmetic mean ratio; aAMR, adjusted AMR; aPTT, activated partial thromboplastin time; INR, international normalized ratio; 95%CI, 95% confidence interval; p, level of significance; VWF, von Willebrand factor.

Table S5.Association of coagulation proteins with NT-proBNP levels in the female group one year after ICU discharge.

Coagulation biomarkers	***Un-adjusted***		***Adjusted***	
	AMR (95%CI)	***p***	***aAMR (95%CI)***	***p***
Factor IX	0.99 (0.97-1)	0.162	0.99 (0.97-1)	**0.024**
Protein C	0.98 (0.97-1)	**0.033**	0.98 (0.97-1)	**0.022**
Protein S	0.99 (0.98-1)	0.096	0.98 (0.97-0.99)	**0.003**
VWF	1.01 (0.99-1.03)	0.393	1.01 (1-1.03)	0.176
aPTT	1 .01 (0.98-1.02)	0.967	0.99 (0.99-1.01)	0.670
INR	1.99 (0.49-8)	0.361	1.06 (0.46-2.44)	0.905

Statistics: Data were calculated using generalized linear models (GLM) with gamma distribution. AMR, 95% confidence intervals (lower boundary: 2.5%, upper boundary: 97.5%) and p-value are shown for both adjusted and unadjusted models. GLMs were adjusted by age, gender, age, gender, follow-up time, body mass index (BMI) and anticoagulant therapy at the end of follow-up. Significant associations are shown in bold. **Abbreviations**: AMR, arithmetic mean ratio; aAMR, adjusted AMR; aPTT, activated partial thromboplastin time; INR, international normalized ratio; 95%CI, 95% confidence interval; p, level of significance; VWF, von Willebrand factor.

Table S6. Association of coagulation proteins at ICU admission with NT-proBNP levels one year after ICU discharge.

Coagulation biomarkers	***Un-adjusted***		***Adjusted***	
	AMR (95%CI)	***p***	***aAMR (95%CI)***	***p***
Factor IX	1.02 (0.97-1.08)	0.461	1.01 (0.96-1.05)	0.779
Protein C	1.01 (0.96-1.06)	0.754	1.03 (0.99-1.07)	0.133
Protein S	0.99 (0.94-1.04)	0.729	1 (0.95-1.05)	0.946
VWv	1.06 (1.01-1.11)	**0.013**	1.04 (1-1.08)	0.056
aPTT	0.96 (0.9-1.01)	0.151	0.96 (0.92-1.01)	0.120
INR	0.99 (0.97-1.02)	0.793	0.99 (0.97-1.01)	0.180

Statistics: Data were calculated using generalized linear models (GLM) with gamma distribution. AMR, 95% confidence intervals (lower boundary: 2.5%, upper boundary: 97.5%) and p-value are shown for both adjusted and unadjusted models. GLMs were adjusted by age, gender, age, gender, follow-up time, body mass index (BMI) and anticoagulant therapy at the end of follow-up. Significant associations are shown in bold. **Abbreviations**: AMR, arithmetic mean ratio; aAMR, adjusted AMR; aPTT, activated partial thromboplastin time; INR, international normalized ratio; 95%CI, 95% confidence interval; p, level of significance; VWF, von Willebrand factor.

Table S7. Association of coagulation proteins at ICU admission with NT-proBNP levels in the male group one year after ICU discharge.

Coagulation biomarkers	***Un-adjusted***		***Adjusted***	
	AMR (95%CI)	***p***	***aAMR (95%CI)***	***p***
Factor IX	1.01 (0.92-1.1)	0.850	0.99 (0.92-1.05)	0.674
Protein C	0.97 (0.91-1.05)	0.478	1.01 (0.96-1.06)	0.822
Protein S	1.04 (0.94-1.14)	0.444	1 (0.93-1.08)	0.979
VWF	1.07 (1-1.14)	0.056	0.99 (0.93-1.05)	0.747
aPTT	1 (0.99-1.01)	0.859	0.99 (0.99-1.01)	0.926
INR	1.87 (0.82-4.24)	0.147	0.90 (0.42-1.95)	0.800

Statistics: Data were calculated using generalized linear models (GLM) with gamma distribution. AMR, 95% confidence intervals (lower boundary: 2.5%, upper boundary: 97.5%) and p-value are shown for both adjusted and unadjusted models. GLMs were adjusted by age, gender, age, gender, follow-up time, body mass index (BMI) and anticoagulant therapy at the end of follow-up. Significant associations are shown in bold. **Abbreviations**: AMR, arithmetic mean ratio; aAMR, adjusted AMR; aPTT, activated partial thromboplastin time; INR, international normalized ratio; 95%CI, 95% confidence interval; p, level of significance; VWF, von Willebrand factor.

Table S8. Association of coagulation proteins at ICU admission with NT-proBNP levels in the female group one year after ICU discharge.

Coagulation biomarkers	***Un-adjusted***		***Adjusted***	
	AMR (95%CI)	***p***	***aAMR (95%CI)***	***p***
Factor IX	1.04 (0.96-1.12)	0.327	1.03 (0.97-1.09)	0.371
Protein C	1.05 (0.96-1.14)	0.304	1.05 (0.98-1.12)	0.182
Protein S	0.98 (0.9-1.06)	0.572	1.03 (0.96-1.1)	0.463
VWF	1.05 (0.99-1.11)	0.148	1.04 (1-1.09)	0.066
aPTT	1 (0.98-1.02)	0.967	0.99 (0.98-1.01)	0.436
INR	1.99 (0.49-8)	0.361	1.36 (0.48-3.91)	0.582

Statistics: Data were calculated using generalized linear models (GLM) with gamma distribution. AMR, 95% confidence intervals (lower boundary: 2.5%, upper boundary: 97.5%) and p-value are shown for both adjusted and unadjusted models. GLMs were adjusted by age, gender, age, gender, follow-up time, body mass index (BMI) and anticoagulant therapy at the end of follow-up. Significant associations are shown in bold. **Abbreviations**: AMR, arithmetic mean ratio; aAMR, adjusted AMR; aPTT, activated partial thromboplastin time; INR, international normalized ratio; 95%CI, 95% of confidence interval; p, level of significance; VWF, von Willebrand factor.
